# Continuous Feature-Based Tracking of the Inner Ear for Robot-Assisted Microsurgery

**DOI:** 10.3389/fsurg.2021.742160

**Published:** 2021-11-19

**Authors:** Christian Marzi, Tom Prinzen, Julia Haag, Thomas Klenzner, Franziska Mathis-Ullrich

**Affiliations:** ^1^Health Robotics and Automation, Institute for Anthropomatics and Robotics, Karlsruhe Institute of Technology, Karlsruhe, Germany; ^2^Department of Otorhinolaryngology, Head & Neck Surgery, University-Hospital Düsseldorf, Düsseldorf, Germany

**Keywords:** tracking, feature-based, microscope, image-processing, inner ear, robotic surgery, cochlea implantation

## Abstract

Robotic systems for surgery of the inner ear must enable highly precise movement in relation to the patient. To allow for a suitable collaboration between surgeon and robot, these systems should not interrupt the surgical workflow and integrate well in existing processes. As the surgical microscope is a standard tool, present in almost every microsurgical intervention and due to it being in close proximity to the situs, it is predestined to be extended by assistive robotic systems. For instance, a microscope-mounted laser for ablation. As both, patient and microscope are subject to movements during surgery, a well-integrated robotic system must be able to comply with these movements. To solve the problem of on-line registration of an assistance system to the situs, the standard of care often utilizes marker-based technologies, which require markers being rigidly attached to the patient. This not only requires time for preparation but also increases invasiveness of the procedure and the line of sight of the tracking system may not be obstructed. This work aims at utilizing the existing imaging system for detection of relative movements between the surgical microscope and the patient. The resulting data allows for maintaining registration. Hereby, no artificial markers or landmarks are considered but an approach for feature-based tracking with respect to the surgical environment in otology is presented. The images for tracking are obtained by a two-dimensional RGB stream of a surgical microscope. Due to the bony structure of the surgical site, the recorded cochleostomy scene moves nearly rigidly. The goal of the tracking algorithm is to estimate motion only from the given image stream. After preprocessing, features are detected in two subsequent images and their affine transformation is computed by a random sample consensus (RANSAC) algorithm. The proposed method can provide movement feedback with up to 93.2 μm precision without the need for any additional hardware in the operating room or attachment of fiducials to the situs. In long term tracking, an accumulative error occurs.

## 1. Introduction

Otologic microsurgery requires the surgeon to work at the limit of their visuo-tactile feedback and dexterity. The procedure of a cochlea implantation, for example, consists traditionally of a manually drilled, nearly cone-shaped access beginning on the outer surface of the skull with a diameter of around 30 mm and tapered to a 2 mm narrow opening to the middle-ear (posterior tympanotomy). After visualization of the round window, the cochlea can be opened through the round window or a cochleostomy, an artificial opening drilled by the surgeon. The surgeon then has to move a 0.3–1 mm thin electrode array through the posterior tympanotomy in the even more narrow cochlea. Robotic systems can exceed human precision in order of multiple magnitudes. Therefore, it is obvious that otologic microsurgery can highly benefit from robotic assistance.

When introducing novel technological robotic aids into surgery, space is often a critical factor. The closer to the surgical situs, the more important it is to keep the spacial obstruction to a minimum. In microsurgical interventions, a surgical microscope is always present. Therefore, mounting an assistive robotic manipulator to a microscope's optic unit poses high potential for robotic support. This allows for bringing the robot into close proximity of the situs while maintaining obstruction to the surgeon on a similar level as in regular microsurgery.

While being widely established for ablation of soft tissue (for example in ophthalmology), robotic laser surgery is gaining increasing interest in ablation of bone. In otologic surgery different kind of handheld lasers are used to penetrate the footplate of the stapes and more recently robotic guided lasers for interventions in the inner ear are taken into clinical trials (1). Also ablation of larger volumes of bone tissue could be demonstrated to be ready for clinical applications as for example by AOT's recent certification of CARLO (2), a laser osteotome mounted on a collaborative robot arm. The latter is applied in craniofacial surgery and provides cleaner cuts as well as additional freedom in cut geometry. Also the research project MIRACLE (3) aims on ablation of bone. However, in this case a minimally invasive robotic approach is pursued to reduce trauma. In addition, interventions at the inner ear are in focus of laser ablation of bone (4–6). In combination with sensory feedback about residual bone tissue, laser ablation provides a precise tool for opening of the cochlea. Such robotic systems in particular, could greatly benefit from integration into a surgical microscope toward clinical translation.

However, integration into a movable microscope will pose the challenge for the robotic system to maintain precise registration to the patient. Modern microscopes provide robotic support with position encoders as well as interfaces to marker based registration systems (7). Still, registration may be interrupted or become inaccurate by small, sudden movements, which can occur due to unintended contact with the microscope or movement of the patient. Compensating for such motions will be a necessary skill for any microscope-mounted robotic system manipulating tissue.

Modern surgical microscopes provide a magnified image of the surgical scene and integrate cameras or adapters for camera attachment. Often, the recorded images can be streamed to monitors in the operation room (OR) by standardized interfaces. Thus, the magnified image provides information available at no additional cost of hardware. Utilizing these images to derive movement information for a robotic system would thus be integrateable without increased efforts. In addition, such an image based tracking system would gain precision from the microscopes magnification.

State of the art for tracking the surgical microscope (and other tools or the patient) remain retro-reflective markers detected optically by infrared (IR) cameras in combination with IR-LED (7). Recent works have focused on using features based tracking in microscopes images for augmentation and registration of preoperative data. For example in (8), the pose of the cochlea is augmented for navigation support. Here, *Speeded Up Robust Features* (SURF) were used for maintaining the augmented images registered. In (9), the tips of the instruments for microsurgical intervention had to be colored green to allow for pose estimation through the microscope's image.

Extending the modification of tools or introduction of fiducials, this work aims on processing the microscope images based on natural features to gather information of the relative movement between the microscope and the patient. These tracking information can then be made available to enable robotic assistance. Cochleostomy is used as an example for a common and standardized intervention with high potential for automation.

## 2. Materials and Methods

### 2.1. Imaging Setup

The investigated method aims on interfering as little as possible with the existing surgical workflow. This also means no additional hardware should be introduced into the operating room or, in particular, in proximity to the patient. Therefore, the existing imaging capabilities of commercial surgical microscopes should be utilized. To record microscope images, most conventional microscopes are equipped with standardized flanges to attach a camera as it is often used for documentation in current practice. Here, a computer with a frame grabber (DeckLink Recorder Mini 4K, Blackmagic Design Pty Ltd, Victoria, Australia) is used to gain access to the image frames. The processing computer is equipped with an Intel(R) Core(TM) i7-8086K CPU and GeForce GTX 1080 Ti GPU. These components are the only additional hardware, which could be easily positioned outside of the OR.

### 2.2. Image Processing Pipeline

#### 2.2.1. Framework

To facilitate data exchange and enable a connection to a future robotic system the Robot Operation System (ROS, Distribution *Noetic*) is used as a software framework. A ROS driver for the frame grabber was developed to provide the images from the microscope to ROS. The raw frames are submitted to the image processing node on a ROS topic. For representation of pose information, ROS' dedicated data structure called *TF-Three* is used. It represents pose information in a hierarchical structure and is easily expandable and accessible in a network.

#### 2.2.2. Scene Tracking

**Figure 1:**
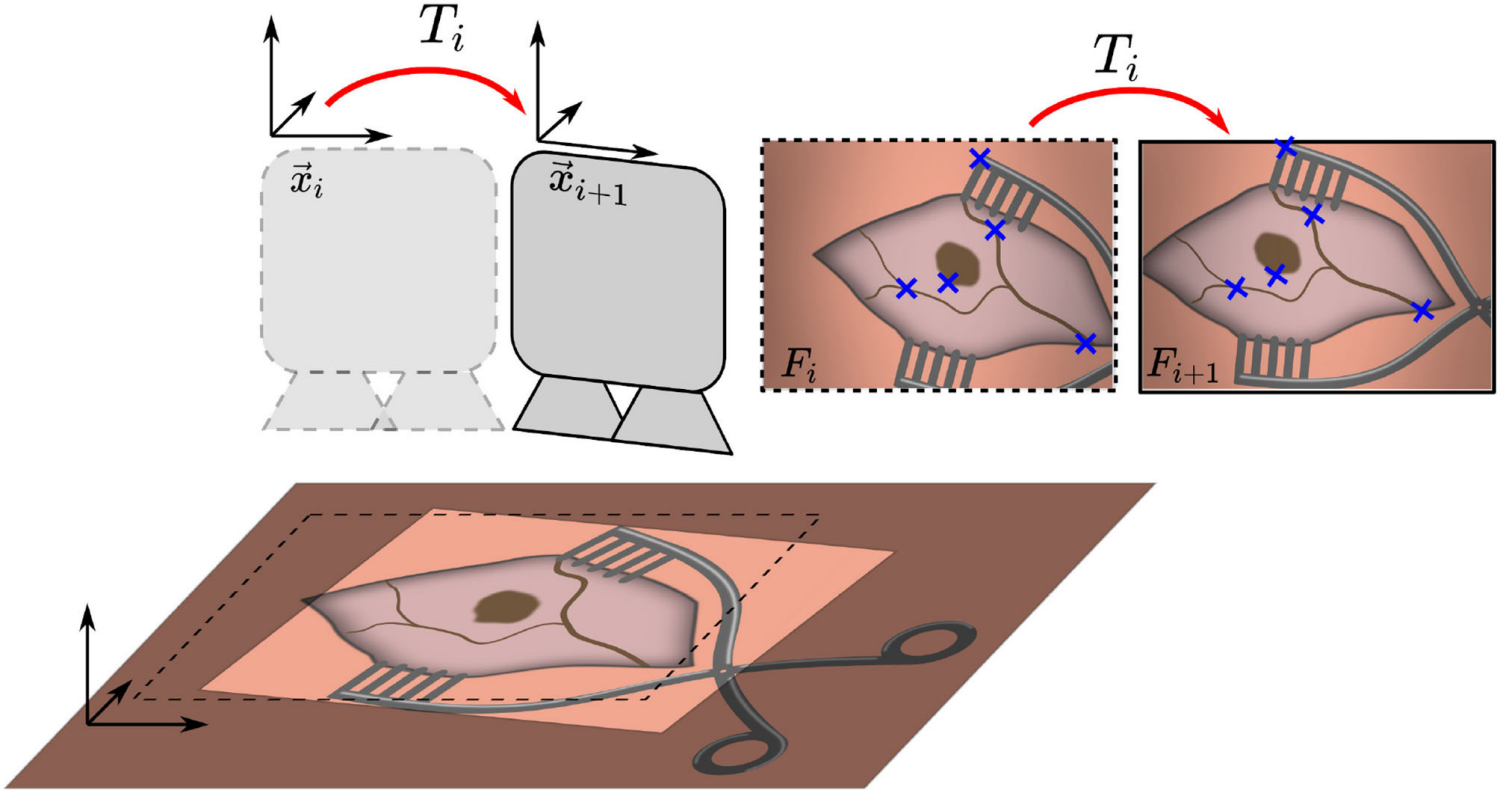
Overview of the proposed method. Two consecutive microscope image frames *F*_*i*_ and *F*_*i*+1_ are processed to identify features, which are used to estimate the transformation *T*_*i*_ and maintain an initial registration by updating position *x*_*i*_ to *x*_*i*+1_.

**Figure 2:**
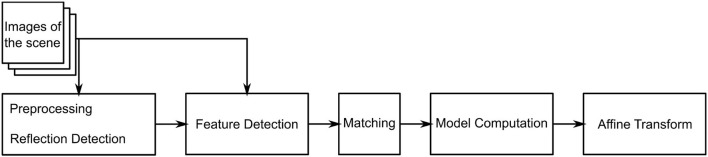
UML diagram of the proposed algorithm.

**Figure 3:**
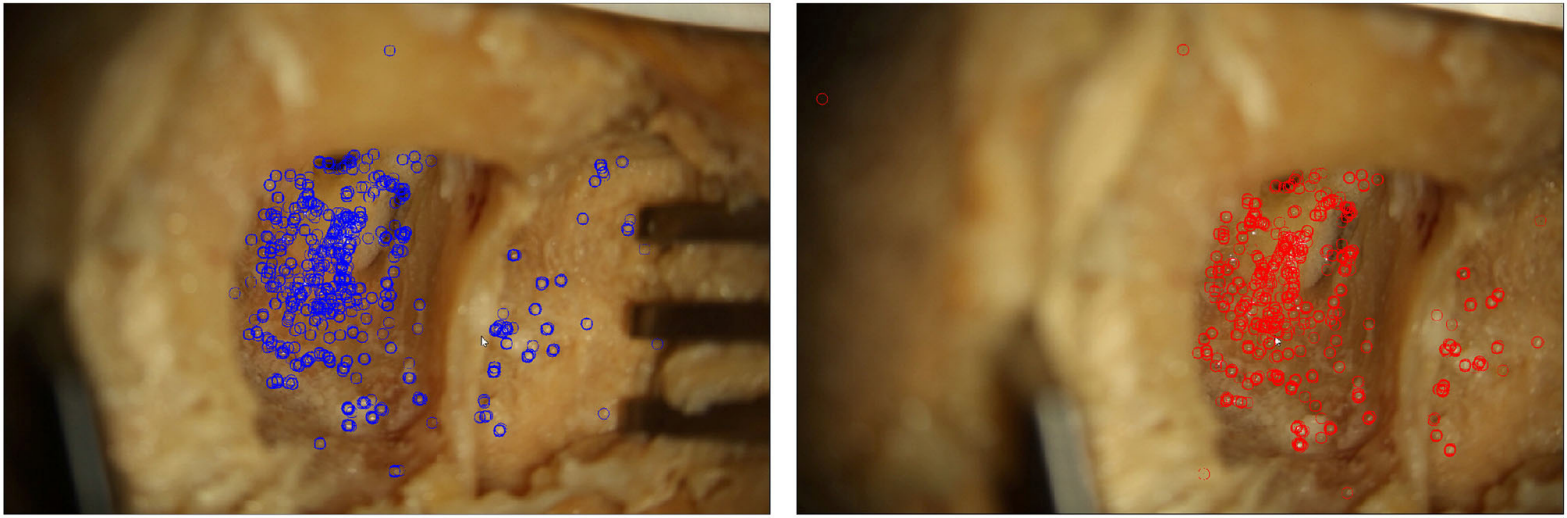
Two microscope images of a moving situs. In each frame, features detected by the proposed algorithm are marked.

Due to the bony structure of the surgical site, the cochleostomy scene is assumed to move rigidly and tissue deformations can be neglected. Movement is tracked in 2D in the microscope image plane, as illustrated in Figure 1. The proposed tracking algorithm provides an estimate of the relative motion between the surgical situs and microscope, given only the microscope's image stream and no further information. Motivated by microscope mounted robotic systems, this information would be sufficient to allow for compensation of unintended motion of either patient or microscope. In the proposed method, two subsequent images are compared and their affine transformation


(1) $$\begin{array}{l} T=\left[\begin{array}{ccc} a_{00} & a_{01} & b_{0}\\ a_{10} & a_{11} & b_{1}\\ 0 & 0 & 1 \end{array}\right] \end{array}$$

is estimated. The algorithm consists of three main steps. The flowchart in Figure 2 outlines the algorithm. First, a feature detection algorithm (see section 2.2.4) identifies distinct natural landmarks. Second, the identified features are matched. An example of these identified features is displayed in Figure 3. Third, a transformation model between the established matches is estimated. Additional preprocessing to detect reflection artifacts in the images can increase tracking robustness for some surgical scenes. Here, thresholding is used to confine illumination artifacts in the field of view. The full image processing pipeline is illustrated in Figure 2.

#### 2.2.3. Image Preprocessing

Lighting-dependent artifacts appear as pixels with distinctively high color values in the microscope image. This delimits the affected points from their neighboring points. Accordingly, thresholding is a reasonable approach for reflection detection (10). As reflections are often prone to wrongly serving as detected features, thresholding is conducted before feature detection. It is conducted for each pixel comprising a saturation *S* and intensity *I*. If the statement in Equation (2) holds true, the pixel is added to the mask.


(2) $$\begin{array}{l} I<\tau_{1}\cdot I_{\text{max}}\cup S>\tau_{2}\cdot S_{\text{max}} \end{array}$$

Here, *I*_max_ is the image's maximum intensity and *S*_max_ the image's maximum saturation. Parameters τ_1_ and τ_2_ are the respective thresholds, which were iteratively identified and evaluated. Sufficiently suitable values are given by τ_1_ = 0.8 and τ_2_ = 0.2. Preprocessing generates a mask, which excludes part of the images from further processing.

#### 2.2.4. Feature Detection

The masked image is used to detect features utilizing the *Oriented FAST and Rotated BRIEF* (ORB) algorithm first presented by (11). It was developed as an alternative to the patented *Scale Invariant Feature Transform* (SIFT) algorithm (12). ORB is faster than SIFT and other alternatives like SURF, while being more sensitive to movements and more robust (13). The ORB feature detector is invariant to translation, rotation and scaling of the image, as well as robust against illumination changes and noise. The first step of the ORB algorithm is the detection of keypoints. These are generated by the *Features from Accelerated Segment Test* (FAST), which are combined with an orientation measure. For all keypoints found, a Binary Robust Independent Elementary Features (BRIEF) descriptor is computed. The number *k* of desired keypoints depends on the size of the obtained images. For 1080p images, *k* is suggested to be set to 2,000 according to the results by (14). Here, the ORB algorithm is implemented in Python using the image processing library OpenCV (15).

#### 2.2.5. Transformation Model

Natural landmark detection results in a set of keypoints and their descriptors. Given two such sets obtained from images that share image features, the next step in our tracking algorithm is to find the corresponding matches between two images based on the detected features. The found matches are then used to estimate the affine transformation between these scenes. Since surgical scenes do not vary significantly in color or features it is likely that many keypoints are matched incorrectly despite the computed descriptors. Thus, a model estimation algorithm that is robust against a high ratio of mismatches (outliers) is required. The Random Sample Consensus (RANSAC) algorithm (16) estimates a model's parameters based on a set of data *D* which contains more points than are required for model description.

The desired model is the affine transformation *T* (see Equation 1). The set *D* is formed by tuples of ORB features with matching BRIEF descriptor in two subsequent images. The model is estimated to approximate the best affine transformation with respect to the translations of the features. For the developed image processing software, the implementation of RANSAC from the Python library *scikit-images* (17) was used. The affine transformations *T*_*n*_ of each iteration *n* can be cascaded to form an accumulated position ${\vec{x}}_{n}$ and the measured trajectory (formed by all *x*_*i*_ ∈ {1, …, *n*}) of the relative movement of situs and microscope.


(3) $$\begin{array}{l} {\vec{x}}_{n}=\left(\prod^{n}T_{n}\right)\;(\begin{array}{c} 0\\ 0\\ 1 \end{array}) \end{array}$$

The evaluated position is passed to the TF-tree in ROS to easily be accessible by any connected robotic system.

### 2.3. Experimental Evaluation

For evaluation of the proposed algorithm, a robot is used to create a precise reference movements of a specimen. The trajectories are captured through the microscope and the image processing pipeline estimates the movement. Comparing estimated movement and reference movement allows for determination of a tracking error.

**Figure 4:**
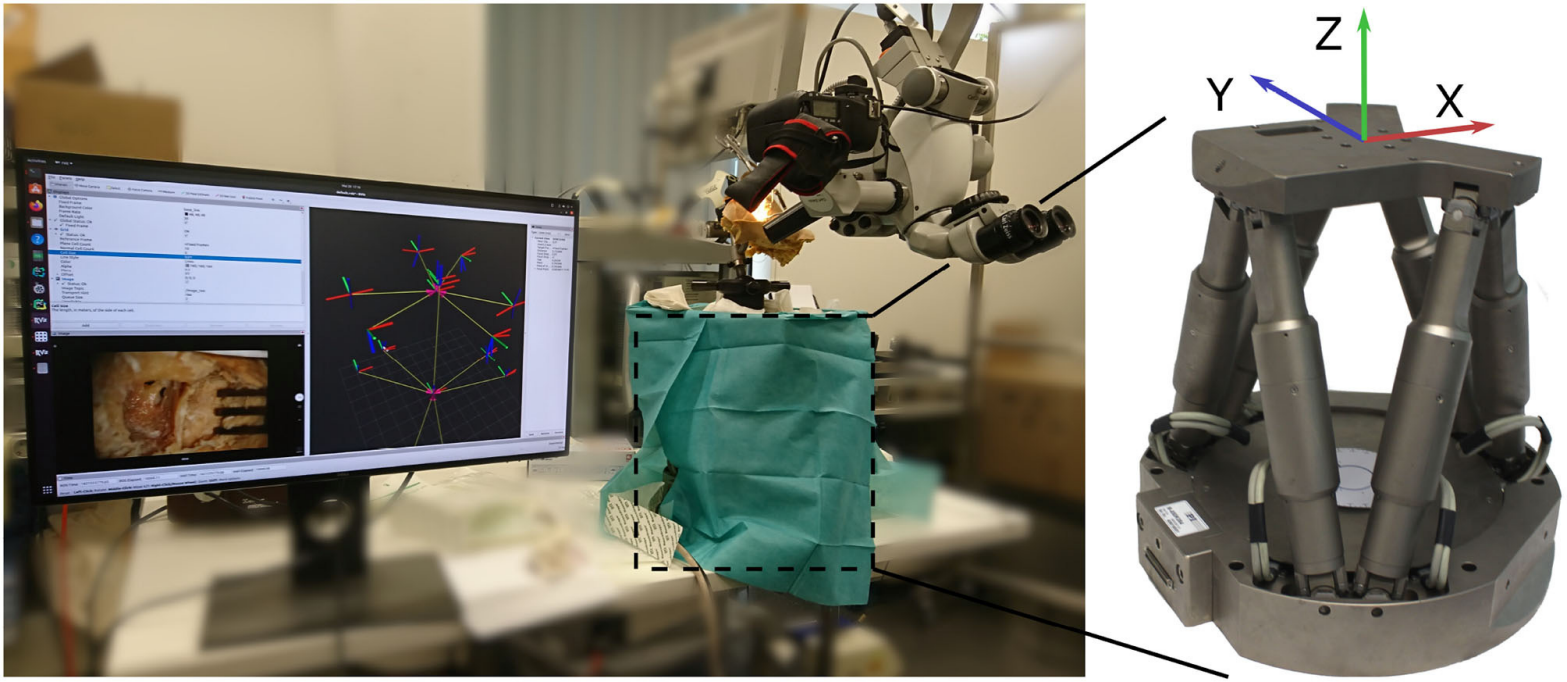
Overview of the evaluation setup. A camera is attached to the side port of a surgical microscope. Below, the phantom is attached to a Stewart platform (covered by drapes). The robot is used for generating precise reference movement data.

The setup for evaluation consist of a commercial surgical microscope (OPMI Pro Magis/S8, Carl Zeiss AG, Oberkochen, Germany). A camera (Canon EOS 100D) is attached to the side port of the microscope, recording a video stream. The video stream is captured by the frame grabber card in the processing computer. Below the microscope the surgical scene is set up on a Stewart platform (M-850, Physik Instrumente GmbH, Karlsruhe, Germany) that allows for defined control of precise reference movement with a repeatability of 2μm. The robot is controlled by the processing computer using ROS. The complete experimental setup is depicted in Figure 4.

**Figure 5:**
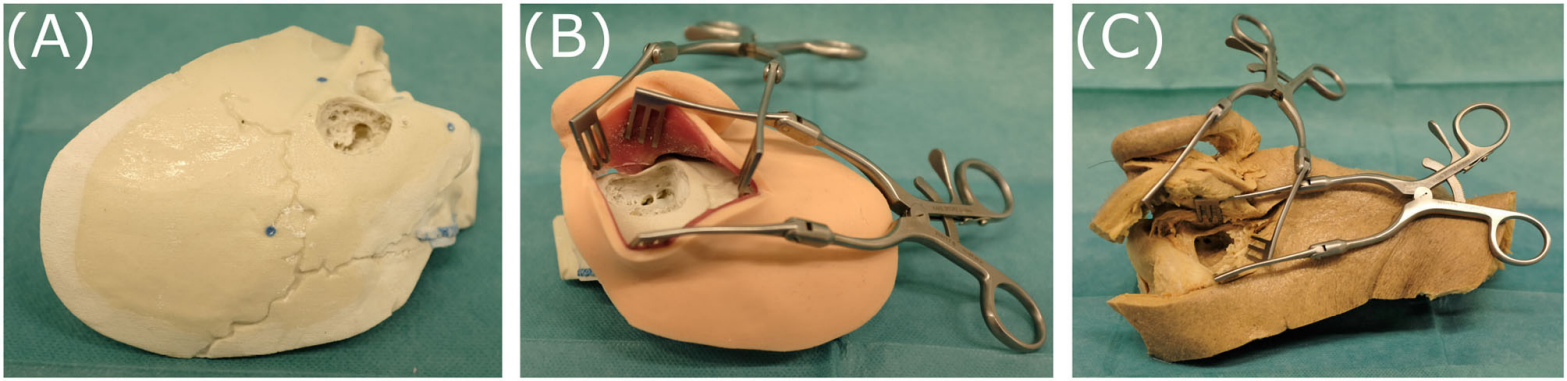
The three specimens evaluated, representing different levels of realism: **(A)** temporal bone phantom (TBP), **(B)** temporal bone phantom with skin-like material (TBPs), **(C)** cadaveric temporal bone (CTP).

To evaluate the presented tracking pipeline on several levels of realism and allow for comparison between different domains, three specimens are evaluated:

1. A temporal bone phantom **(TBP)** (PHACON GmbH, Leipzig, Germany) comprising only of bone-like material (see Figure 5A)
2. A temporal bone phantom comprising of bone-like material covered with multilayered skin-like material **(TBPs)** (PHACON GmbH, Leipzig, Germany). The skin incision is held apart by self-retaining retractors to facilitate good visualization (see Figure 5B)
3. A cadaveric temporal bone **(CTP)**. The skin incision is held apart by self-retaining retractors to facilitate good visualization (see Figure 5C).

All models and phantoms have been prepared to represent the last surgical phase before opening the cochlea. Therefore, a skin incision, mastoidectomy and posterior tympanotomy have been previously performed. The microscope is set up to provide a view similar to visualization during a surgical intervention.

The Stewart platform provides 6 degrees of freedom motion, however only translation movement along its x- and y-axes are used for reference motion (compare Figure 4). For data recording, the x-axis and y-axis of the robot are aligned manually to the image axes.

Motion of the patient is then simulated by driving the robot along a predefined trajectory. First, linear translational movement in x- and y-directions are evaluated. To also cover combinations of x- and y-motion in the 2D image space, we additionally evaluated spiral motion of the robot. The processing of the image data, as well as the control of the robot and sampling of reference data were conducted on the same computer to allow for data synchronization. The data was recorded for later evaluation as *rosbag*, ROS' data recording format. Equations (4) and (5) define the waypoints for the chosen trajectories. As soon as one waypoint was reached by the robot, the next one was passed to the robot's controller. In between the waypoints, the used controller interpolates a linear trajectory. The robot conducted the movement with its maximum speed of 2 mm/s. Linear translations in x- and y-directions (i.e., cross-shape) are defined by the waypoints


(4) $$\begin{array}{l} {\vec{c}}_{lin,n}\in \left\{\left(\begin{array}{c} 0\\ 0 \end{array}\right),\left(\begin{array}{c} 0\\ 10 \end{array}\right),\left(\begin{array}{c} 0\\ -10 \end{array}\right),\left(\begin{array}{c} 0\\ 0 \end{array}\right),\left(\begin{array}{c} 10\\ 0 \end{array}\right),\left(\begin{array}{c} -10\\ 0 \end{array}\right),\left(\begin{array}{c} 0\\ 0 \end{array}\right)\right\}. \end{array}$$

The spiral trajectory is defined by the waypoints


(5) $${\vec{c}}_{spir,n}=\left(\begin{array}{l} 10\frac{n}{50}\sin\left(4\cdot 2\pi \frac{n}{50}\right)\\ 10\frac{n}{50}\cos\left(4\cdot 2\pi \frac{n}{50}\right) \end{array}\right)\;{|}_{\forall }n=[0,25]\in \mathbb{N}.$$

## 3. Results

### 3.1. Frame to Frame Precision

To evaluate the precision of the algorithm, for two consecutive frames the estimated affine transformations is compared to the reference transformation of the robot. For the evaluated trajectories the translation error $\vec{E}$ is calculated by Equation (6) from the translations given from the algorithm $\Delta {\vec{x}}_{\text{ORB}}$ and reference from robot $\Delta {\vec{x}}_{\text{robot}}$.


(6) $$\begin{array}{l} \vec{E}=\Delta {\vec{x}}_{\text{ORB}}-\Delta {\vec{x}}_{\text{robot}} \end{array}$$

**Figure 6:**
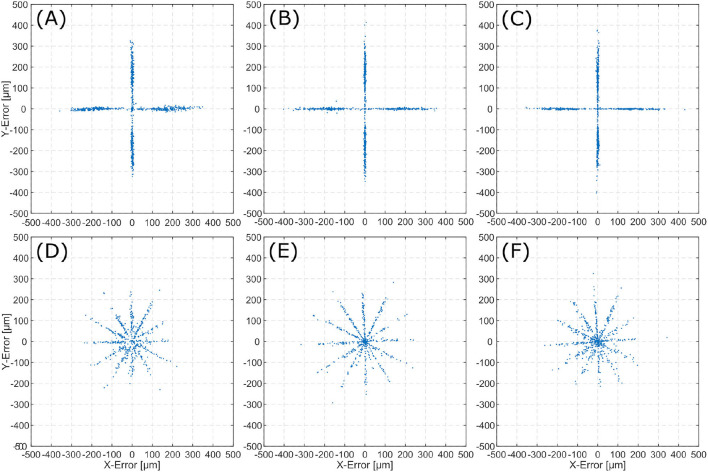
Error ($\vec{E}$) distribution for the evaluated scenes. The top row displays 2D-errors for the linear trajectories on TBP **(A)**, TBPs **(B)**, and CTP **(C)**. The bottom row displays 2D-errors for the spiral trajectories on TBP **(D)**, TBPs **(E)**, and CTP **(F)**.

For each trajectory (linear and spiral), $\vec{E}$ is calculated for all two consecutive frames. This results in an error distribution, which is plotted for each inner ear model. Error distributions are presented for the linear trajectories (Figures 6A–C), and spiral trajectories (Figures 6D,E).

The mean absolute error distance μ is derived from the *n* sets of consecutive frames by


(7) $$\begin{array}{l} \mu =\frac{1}{n}\overset{n}{\sum }||{\vec{E}}_{n}||. \end{array}$$

Table 1 summarizes the mean errors of the tracked motion and their standard deviations for each specimen.

**Table 1:** Summary of the tracking precision results.

	**TBP**		**TBPs**		**CTP**	
	**Mean**	**Std**	**Mean**	**Std**	**Mean**	**Std**
Linear	93.9	118.4	135.8	114.3	110.1	112.6
Spiral	98.7	79.0	97.2	85.9	93.2	80.0

*For each sample and each tested trajectory the mean error for the tracking error of two consecutive frames and their standard deviation are presented (all values in μm)*.

Error distributions in Figure 6 show that the x and y locations of the error correlate with the number of trajectory sections with a constant orientation. Execution of linear trajectories in x- and y-directions results in error aggregation around *x* = 0 and *y* = 0. The spiral trajectories result in error aggregation along distinct angles.

### 3.2. Trajectories

**Figure 7:**
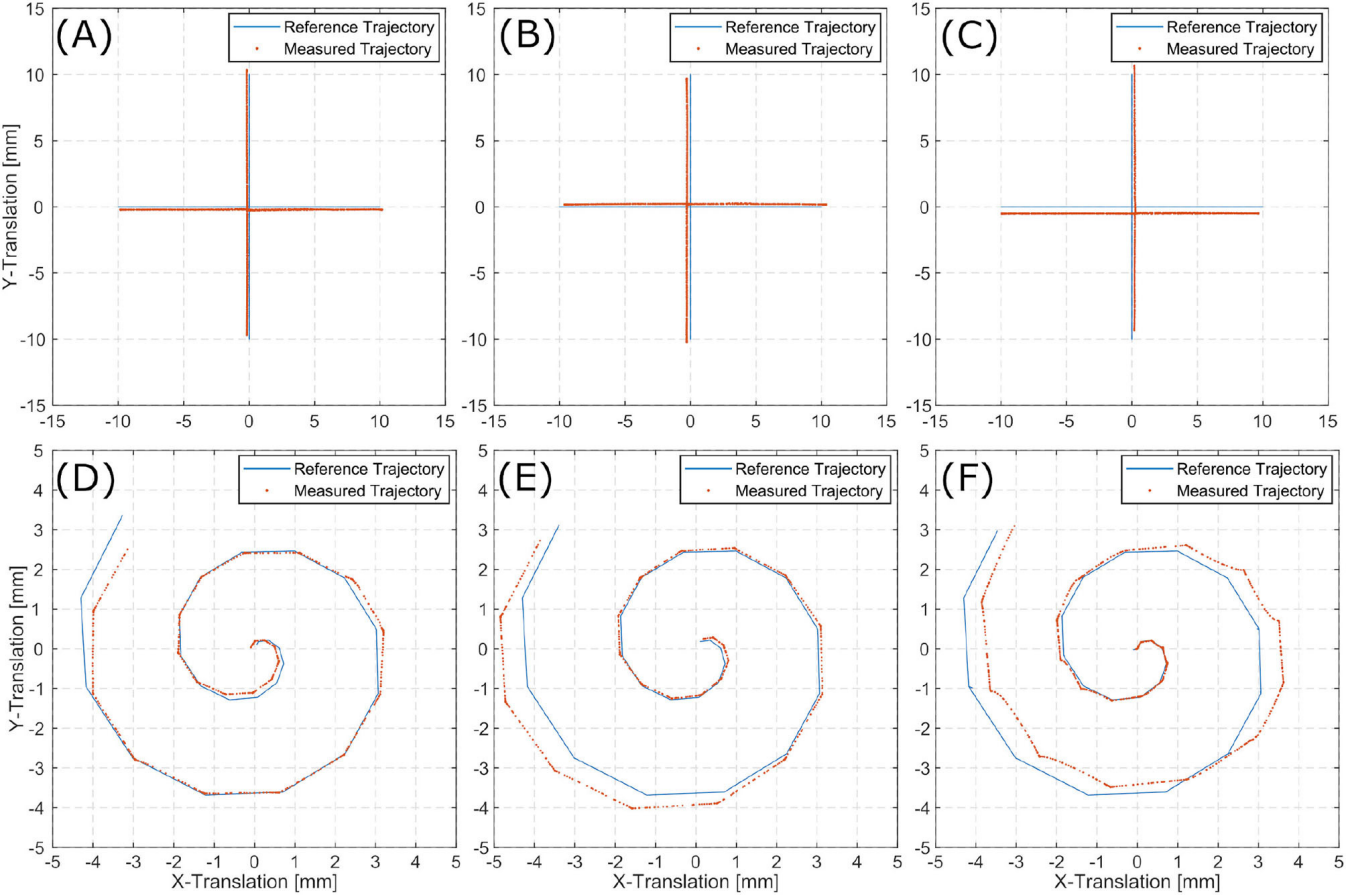
Linear **(A–C)** and spiral **(D–F)** trajectories for TBP **(A,D)**, TBPs **(B,E)**, and CTP **(C,F)**.

A set of affine transformation is estimated from the image stream. Cascading these transformations and applying them to the initial pose, results in an estimation of the current pose. The translational information of these poses can be displayed as the scenes full trajectory. This trajectory allows for comparison to the reference trajectories as executed by the Stewart platform. Figures 7A–C show reference trajectories (blue) and the image based trace of motion (red) for linear trajectories for each inner ear model (i.e., TBP, TBPs, and CTP). Figures 7D–F show reference trajectories and the image based trace of motion for the spiral trajectories for each scene.

The tracked linear trajectories (i.e., cross-shape) display an offset to the reference path but returns to its original starting point for all three models. For the spiral trajectories the accumulating pose error results in a total error of the final position.

### 3.3. Performance

The average duration of each algorithmic step in the process is listed in Table 2. These values refer to the runtime per image for images of size 1,920 × 1,080 and 960 × 540 px. The runtime is measured using 30 random images of the surgical site. The total runtime is listed for the implemented algorithm using scikit's RANSAC implementation, which was used for model estimation in this work.

**Table 2:** Results of performance evaluation.

**Step**	**Runtime mean ± standard deviation in ms**	
	**1,920 × 1,080**	**960 × 540**
Feature detection	74 ± 9	18 ± 1
Matching	16 ± 3	12 ± 8
Model estimation	246 ± 77	234 ± 11
Preprocessing	36 ± 9	17 ± 5
Approximate total time	327	281

*Runtime for 1,920 × 1,080 (Full HD) and 960 × 540 images are compared*.

## 4. Discussion

The precision result exhibit few deviations between the phantoms (TBP, TBPs) and the human model (CTP). This leads to the conclusion, that the proposed method is well-suited for application in surgery independent of the specific domain.

The smallest tissue manipulation necessary for the intervention of cochlea implantation is the 2,000 μm opening to the middle-ear. The average translational error for all trajectories and scenarios (93.2–135.8μm) is more then one magnitudes below. Therefore, the frame to frame tracking proves suitable for supporting the localization of an assistive robotic system.

In the error distribution diagrams in Figure 6 a strong correlation between the error and the direction of movement can be observed. For the linear trajectories erroneous motion only occurred along the x- and y-axes. The respective error distributions exhibit errors along the x- and y-axes. This leads to the conclusion, that the presented algorithm can determine the direction of a translation with significantly higher precision than the magnitude of the same translation. For the spiral trajectories the errors are distributed more evenly. The observation of aggregation along distinct angles (i.e., creating the star-like error distribution), can also be explained by the conclusion of higher angular precision. As the spiral trajectory is interpolated by linear sections, translation occurs section-wise linearly and for each section errors aggregate in the respective direction of translation.

The presented algorithm for reconstruction of the trajectory incrementally traces the current relative pose of microscope to patient. Position information only relies on the last increment of the pose as it is derived from the last two consecutive images. Therefore, it suffers from typical loss in precision over time as errors accumulate. For linear trajectories along x- and y-directions this effect is sufficiently small. However, when combining translation in multiple directions in the spiral trajectories, the accumulated error increases over time. The latter displays an accumulating overall position error in all three inner ear models. Presumably, this observation is caused by the aforementioned uncertainty in distance exceeding the angular uncertainty.

Despite the relatively high pose error after conduction of the spiral trajectories, the proposed method is suitable for extension by initial registration of the scene, which may be marker based or manually conducted. The trajectories evaluated in this work have a longer duration (44 s for the linear, 30 s for the spiral) compared to a shock caused by unintended motion in the OR. Thus, more iterations evaluated, which increase the accumulative error, in contrast to a real surgical scenario. In a robotic intervention such as motivated in section 1, global registration is likely to be considered a necessary prerequisite anyway. As an example, we envision a manual registration of an ablation laser spot before the ablation process. This could for example be conducted by manual input through a joystick. Extending this with the presented method represents a reliable safety measure against short unintended motion of patient or microscope.

The current algorithm and used hardware allows for processing of the microscope images in real-time with approximately 3 Hz. The major limitation is given by the RANSAC algorithm. Here scikit's implementation was used as it offers greater flexibility in implementation however in preliminary studies also an openCV implementation's runtime was evaluated and resulted in a significant reduction in the exection of RANSAC from an average of 246 ms down to 4 ms. This demonstrates the high potential software as well as hardware optimization offers for increasing the frame rates.

The presented method is limited to tracking an initially conducted registration and compensate for small errors occurring over short periods of time. The initial registration is outside the scope of this work as several methods have previously been presented. Initial registration methodologies strongly depend on the intervention and the applied robotic system. The presented method is prone to long term drifts of the pose due to accumulation of errors. As the scene can be expected to display only small and fast changes in pose. A suggested improvement may be to compare the current frame not only to the most recent one but also to past image data like a user defined initial frame or images captured multiple iterations earlier. The estimated transform from these frames can be used to correct a global drift of the tracked pose.

For appropriate integration to a robotic system, a frame rate suitable to the robotics kinematics needs to be reached by optimizing hardware, image resolution and implementation. For high speed (short term) tracking of relative pose changes, the here presented method could be extended by the use of inertial measurement units. However, these would require integration into the robotic system as well as attachment to the patient.

The presented method has been evaluated for feature-based tracking of inner ear models in two dimensions only. Here, we assume planar motion of surgical situs in the microscope image. To extend this method to covering full 6D pose estimation, i.e., three translations and three rotations, the estimated model needs to be expanded to a 3D-Transformation, as in


(8) $$\begin{array}{l} T_{3}=\left[\begin{array}{cccc} a_{00} & a_{01} & a_{02} & b_{0}\\ a_{10} & a_{11} & a_{12} & b_{1}\\ a_{20} & a_{21} & a_{22} & b_{2}\\ 0 & 0 & 0 & 1 \end{array}\right] \end{array}$$

For application in clinical intervention the surgical scene might become less rigid for example due to moving instruments (robotic or manual). The same issue is likely to occur for manipulations of the surgical field, obstructions by blood or residual tissue from drilling. If these artifacts only cover small areas of the field of view they are likely to be filtered by the RANSAC algorithm. Future work could investigate the robustness of the presented algorithm against such artifacts. Further approaches could research the masking of instruments and residual tissue in the image before feature detection to avoid falsely using features on the tools instead of the situs for tracking. This challenge could be solved by semantic segmentation of the instruments prior to executing the tracking algorithm, as demonstrated in Bodenstedt et al. (18) for laparoscopic scenes. With sufficient training data, typical instruments are masked from the scene and only the situs' image information are utilized for tracking.

## 5. Conclusion

A method for feature-based tracking of the inner ear for compensation of unintended motion was proposed. It is motivated by its use as safety feature enabling microscope mounted medical robotic assistance. Aiming for application in various fields of microsurgery, the application in cochlea implantation was regarded exemplary. Images from a surgical microscope are processed to derive pose changes between patient and microscope. These information can serve as input for compensating motion of a microscope mounted robotic system. Two consecutive images are analyzed for ORB features, which are matched and an affine transformation is estimated by a RANSAC algorithm. The transform is published in the Robot Operating System for integration into robotic systems. Making use of existing hardware in the OR during microsurgery, the microscope image stream is available for processing without introduction of additional hardware. This potentially allows for simple clinical translation of the proposed method. Evaluation showed sub-millimeter accuracy for frame to frame pose changes but revealed increasing offset in absolute pose due to accumulating errors. Application as shock countermeasure seems promising, however, clinical translation will require extension to 3D tracking and optimized performance.

## Data Availability Statement

The original contributions presented in the study are included in the article/supplementary material, further inquiries can be directed to the corresponding author/s.

## Ethics Statement

The studies involving human participants were reviewed and approved by Ethikkommission an der Med. Fakultät der HHU Düsseldorf Moorenstr. 5 D-40225 Düsseldorf FWA-Nr.: 00000829 HHS IRB Registration Nr.: IRB00001579. The patients/participants provided their written informed consent to participate in this study.

## Author Contributions

CM and JH conceived and implemented the algorithm. CM, TP, and JH conceived, designed, and executed the experimental study. CM, TP, TK, and FM-U analyzed and involved in interpretation of data and made final approval of the version to be published. CM, TP, and FM-U drafted the article. All authors contributed to the article and approved the submitted version.

## Conflict of Interest

The authors declare that the research was conducted in the absence of any commercial or financial relationships that could be construed as a potential conflict of interest.

## Publisher's Note

All claims expressed in this article are solely those of the authors and do not necessarily represent those of their affiliated organizations, or those of the publisher, the editors and the reviewers. Any product that may be evaluated in this article, or claim that may be made by its manufacturer, is not guaranteed or endorsed by the publisher.
